# The impact of sports brand image and product quality on consumer satisfaction: the mediating effects of self-image congruence

**DOI:** 10.3389/fpsyg.2026.1778226

**Published:** 2026-07-13

**Authors:** Junchen Fan, Yajing Fan, Qing Li, Shuhan Yu, Chenjie Liang, Muhammad Ibrar Ahmad, Yi Zhang, Yunhang Lu

**Affiliations:** 1 School of Physical Education and Sports Science, Soochow University, Suzhou, China; 2 Department of Physical Education, Kyungpook National University, Daegu, Republic of Korea

**Keywords:** brand image, consumer satisfaction, mediating effect, product quality, self-image congruence

## Abstract

**Objectives:**

Consumer satisfaction (CS) is a crucial indicator that influences sports brand marketing, yet research examining it from the perspective of consumer self-image congruence (SIC) remains limited. This study focuses on sports brands to explore the effects of brand image (BI) and product quality (PQ) on CS, while testing the mediating role of consumer SIC in the relationship between these factors and satisfaction. This work aims to provide empirical evidence for sports brands to enhance CS and refine brand strategies.

**Methods:**

Questionnaires distributed both online and offline with random sampling were used to collect data from 534 Chinese sports brand consumers. The measurement tools included the Consumer Satisfaction Scale, the Self-Image Congruence Scale, the Brand Image Scale, and the Product Quality Scale. Descriptive and correlation analyses were conducted using SPSS 27.0, while confirmatory factor analysis (CFA) and structural equation modeling (SEM) were performed using AMOS 24.0.

**Results:**

Significant positive correlations existed among BI, PQ, SIC, and CS (*p* < 0.01), and model fit was satisfactory (CMIN/DF = 2.793, RMSEA = 0.058, CFI = 0.930, TLI = 0.922, IFI = 0.930). Path analysis further revealed that BI and PQ significantly and positively predicted SIC and CS, while SIC significantly and positively predicted CS (all paths *p* ≤ 0.001). Mediational analysis indicated that SIC partially mediated the BI→CS relationship (indirect effect = 0.141, 95% CI [0.082, 0.226], accounting for 44.90% of the total effect). Furthermore, SIC partially mediated the relationship between PQ and CS (indirect effect = 0.192, 95% CI [0.108, 0.300], accounting for 34.66% of the total effect).

**Conclusion:**

Both sports BI and PQ significantly enhance CS, with consumer SIC partially mediating the effects of BI and PQ on satisfaction. Therefore, sports brands should simultaneously focus on improving PQ and shaping BI that align with the self-image of target consumer groups to enhance satisfaction.

## 1 Introduction

Consumer satisfaction (CS) plays a crucial role in brand product sales and is regarded as a key factor in a brand’s long-term success (Cooil et al., 2007; Rehman et al., 2025). Consumer satisfaction refers to the comprehensive evaluation and perception consumers hold regarding a product’s quality, performance, value, and overall experience after purchase or use. It represents consumers’ response to whether a product or brand meets their expectations. When product performance exceeds expectations, CS increases; when it falls short, satisfaction decreases (Oliver, 1997). With the rapid expansion of the sports product market, understanding consumer satisfaction has become increasingly important for sports brands.

The global sports products market is projected to grow at a compound annual growth rate (CAGR) of 7% from 2023 to 2027, with the market size reaching US$400 billion in 2024 (Hua Yuan Securities, 2024; Haitong International, 2025). This vast sports consumption market not only exerts significant positive impacts on the economy, employment, and tax revenue but also provides consumers with diverse sports products and experiences, thereby stimulating purchasing behavior (Gao et al., 2022; Kim et al., 2023). Increased consumer spending inevitably leads to evaluations of satisfaction with sports brands. When consumers are satisfied with a specific sports brand, they are more likely to repurchase its products. Conversely, dissatisfaction drives consumers to seek alternatives. Sports brands strive to understand customer behavior given that high satisfaction yields numerous benefits, including enhanced brand reputation and reduced customer churn (Semrad, 2024). In summary, CS fosters repeat purchase intent, thus making significant contributions to sports brand economic growth and substantial market share. Therefore, studying CS holds considerable importance.

Brand image (BI) refers to consumers’ overall perception of a brand, reflecting the information they gather through brand product exposure and promotional activities, the emotional value they perceive, and the brand’s distinctiveness (Gardner and Levy, 1955; Keller, 1993). Since the concept of “brand image” was introduced, academic definitions have varied widely, and each definition is constructed based on different theoretical dimensions. For example, Dobni and Zinkhan (1990) noted that “brand image is essentially a subjective and perceptual phenomenon, its formation dependent on consumers’ rational or emotional interpretations.” This definition precisely reflects the consumer cognitive process. Therefore, the formation of BI largely depends on consumers’ psychological perceptions of the brand. Aaker (2012) defines BI as “a set of cognitively organized associations,” while Keller (1993) describes it as “the brand perceptions reflected in consumers’ memory of brand associations.” Both definitions reveal a common truth: Consumers may hold multiple abstract characteristics about a brand, encompassing both objective attributes and subjective perceptions. Low and Lamb (2000) further elaborate on this concept by stating that BI “is the dual cognitive construct of rational and emotional perceptions consumers form toward a specific brand.” In line with the abovementioned findings, the concept of sports BI is multidimensional, influenced by numerous factors including cognition, emotion, symbolism, and values. It encompasses not only the image of the sports brand’s products themselves but also the public image of the entire sports brand enterprise and the image of its brand services (Malik et al., 2012; Wijaya, 2013).

Product quality (PQ), an equally crucial factor influencing CS, is defined as the product’s ability to perform its intended functions, encompassing durability, reliability, convenience, and other attributes that reflect its value (Garvin and Quality, 1984; Garvin, 1987; Zeithaml, 1988). The literature reveals that reliable PQ directly enhances consumer experience. For instance, research on coffee brands indicates that improving PQ effectively boosts sales, significantly and positively influences consumer purchasing decisions, and helps maintain brand loyalty (Rosanti and Salam, 2021). When a brand’s PQ fails to meet consumer expectations, satisfaction declines (Cadotte et al., 1987). Within sports brands, PQ typically encompasses attributes, such as functionality, materials, comfort, and durability. Research also indicates that PQ plays a crucial role in athletic performance by not only influencing physical capabilities but also providing a degree of protection for users (Claussen et al., 2022; Di Domenico and Collet, 2022). At present, given that sports brand products are being increasingly integrated into our daily lives, PQ has become even more critical. The quality of these products is also correlated with consumer purchase intent (Bashiri et al., 2025).

Although brand image may include some product-related associations, it should not be treated as fully equivalent to product quality (Keller, 1993). Brand image primarily reflects the broader symbolic, affective, and reputational meanings attached to a brand, whereas product quality more directly captures consumers’ evaluations of a product’s functional performance, reliability, and overall excellence. Recent research supports maintaining this distinction. A systematic literature review by Tahir et al. (2024) identified product quality as a key determinant influencing the relationship between brand image and customer satisfaction, suggesting that product quality provides explanatory value beyond brand image alone. Empirical findings also indicate that the two constructs do not play interchangeable roles. For example, Veas-González et al. (2024) found that product quality was a crucial driver of consumer satisfaction and loyalty, whereas brand image did not significantly moderate the satisfaction–loyalty relationship, implying that favorable brand perceptions cannot substitute for evaluations of actual product performance. Similarly, Jailos and Magasi (2025) modeled product quality and brand image as separate independent variables and found that both exerted significant positive effects on customer outcomes. Therefore, recent evidence suggests that BI and PQ should be treated as analytically distinct constructs rather than being collapsed into a single dimension.

The concept of “self-image congruence” (SIC) originated from the theory of “self-concept,” which refers to an organized whole of an individual’s perceptions, beliefs, and evaluations of themselves (Rogers, 1951). Since self-concept began attracting the attention of consumer behavior researchers at the beginning in the 1960s, it has become a significant theoretical perspective employed in consumer behavior studies (Birdwell, 1968). Consumer self-consistency refers to consumers’ overall perception and understanding of themselves as objects, including their recognition and evaluation of their own characteristics, values, lifestyles, and social roles. SIC theory posits that people generally act in ways that maintain and enhance their self-concept, comparing BIs with their self-image during consumption. Self-image is described as the multifaceted cognitive framework individuals hold about themselves (Sirgy, 1982). This involves treating oneself as an object of observation, thereby encompassing the overall perception and understanding of one’s traits, values, lifestyle, and social roles. Research further indicates that SIC may influence consumer behavior and purchasing choices (Hosany and Martin, 2012).

Although previous research has extensively explored the impact of brand image and product quality on consumer satisfaction, few studies have explained why these relationships need to be integrated into a single theoretical framework within the context of sports brands (Pradita and Sitio, 2020; Araújo et al., 2023). Sports brands encompass both BI as a reflection of symbolic value and product quality as an indicator of functional value (Park et al., 1986). Consumers do not evaluate these cues in isolation but are likely to integrate and interpret them through self-image congruence. In this regard, self-image congruence is not merely an additional variable but a crucial psychological mechanism linking external brand-related cues to consumer satisfaction. Therefore, the present study focuses on sports brands to investigate the mediating effect of consumer SIC between sports BI, PQ, and CS.

## 2 Literature review and hypothesis development

### 2.1 Brand image and consumer satisfaction

Brand image, representing consumers’ perceptions and feelings toward a brand, directly influences their product perceptions and expectations, thereby affecting satisfaction. Consumers tend to favor positive BIs, which in turn enhance trust and strengthen purchase intent (Tahir et al., 2024). Conversely, negative brand images may lead to consumer attrition. Satisfaction, as an emotional state of consumers, is a crucial factor influencing their willingness to purchase a brand’s products (Mainardes et al., 2023). From a service industry perspective, a strong connection exists between BI and CS; in particular, enhancing BI increases consumer recognition of the brand. Possessing a memorable and positive BI significantly elevates CS (Dam and Dam, 2021). Similar findings exist in e-commerce. For instance, a study examining the relationship between BI and CS in Indonesian e-commerce revealed a significant positive correlation between them (Handayani et al., 2021). A study on badminton racket brands (Yonex users) found that a positive brand image not only enhances consumers’ favorable evaluations of sports brand products but also helps increase their satisfaction levels (Jafar and Aziz, 2024). In line with the above information, the present study proposes the following hypothesis for athletic brands:

**H1**: A positive athletic BI positively influences CS.

### 2.2 Product quality and consumer satisfaction

Product quality refers to the characteristics and capabilities of a product, its ability to meet consumer needs and expectations, and the extent to which it meets or exceeds these standards. PQ encompasses multiple dimensions, including functionality, durability, reliability, and aesthetic design. Hence, it is not merely a technical standard but is also closely related to consumers’ subjective experiences (Stone-Romero et al., 1997). Hanaysha and Abd Ghani (2016), Diputra and Yasa (2021), achieved significant findings in the automotive industry and Samsung smartphone industries, respectively, with both studies revealing that superior PQ significantly impacts CS. Shifting focus to sports brands, Ristevska-Jovanovska (2025) highlights that PQ influences consumer perceptions when selecting sports brands, necessitating high-quality offerings to meet consumer demands in competitive markets. Based on the above information, H2 is proposed:

**H2**: Positive PQ positively influences CS.

### 2.3 Mediating role of consumer self-image congruence

Sirgy et al. (2016) introduced self-consistency theory and defined self-consistency as the subjective experience arising from the interaction between consumers’ self-concepts and the image of product users during the purchasing process. According to this research, when consumers’ self-concepts align with brand personalities, they tend to develop strong purchase intentions. In consumer behavior studies, self-consistency theory measures brand–consumer fit. When purchasing athletic brands, consumers compare BI, PQ, personal image, and individual athletic preferences to identify matching brands (Sirgy et al., 1997).

Self-image congruence has become a significant theoretical perspective in consumer behavior research. In the context of sports brands, consumers first interpret these external brand-related cues in terms of personal meaning and self-relevance. A favorable brand image may signal a lifestyle, personality, or social image with which consumers identify, while high product quality may indicate that the product fits their standards, usage habits, and desired self-presentation. Through this process, consumers evaluate whether the brand is congruent with who they are or who they aspire to be. Satisfaction is therefore shaped not only by the positivity of brand cues themselves, but also by the extent to which those cues are integrated into consumers’ self-concept. In this sense, self-image congruence functions as a psychological mechanism through which brand image and product quality are translated into satisfaction evaluations. Specifically, consumers assess the degree to which sports brand products align with their self-image, thereby selecting sports brand products that better match their self-image and yield higher levels of satisfaction. In a study of supermarket shoppers, self-image congruence emerged as a significant mediating factor, demonstrating that self-congruence fosters strong consumer attachment, satisfaction, and loyalty toward a brand, and that it helps maintain consumers’ self-identity (Wakaba et al., 2024). In another study on green brands, self-consistency similarly served as a primary mediating factor, indicating that when selecting green brand products, consumers seek eco-friendly brands that align with their self-identity, which significantly influences their purchase intention (Chan, 2025). These studies found that SIC, as a subjective perception of the self and the degree of alignment between personal image and BI, significantly influences CS. On the basis of this discussion, the present study proposes Hypotheses 3 and 4:

**H3**: Self-image congruence mediates the relationship between sports BI and CS.

**H4**: Self-image congruence mediates the relationship between PQ and CS.

**FIGURE 1:**
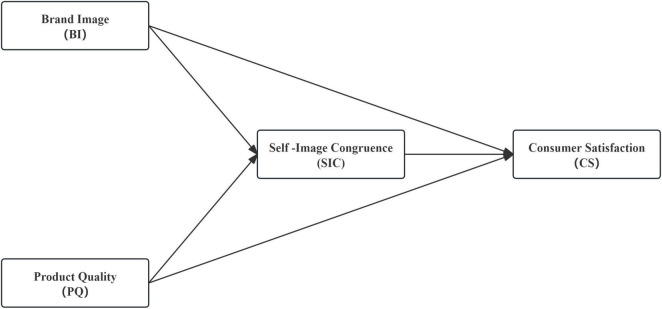
Hypothesized mediation model among brand image, product quality, self-image congruence, and consumer satisfaction.

Thus, brand image and product quality are important antecedents of consumer satisfaction. Moreover, consumers’ evaluations of sports brands are shaped not only by symbolic perceptions and functional judgments, but also by the extent to which the brand is perceived as congruent with their self-image. Accordingly, the present study proposes that brand image and product quality positively influence consumer satisfaction, and that self-image congruence mediates these relationships in the context of sports brands. Therefore, four hypotheses are developed to test the direct effects of brand image and product quality on consumer satisfaction, as well as the mediating role of self-image congruence. The hypothesized mediation model is presented in Figure 1.

## 3 Materials and methods

### 3.1 Participants

Random sampling was employed, and data collection was conducted using questionnaires distributed both online and offline. The participants were sports brand consumers across different age groups, occupations, and genders living within Jiangsu Province. The participants were asked to assess certain sports brands based on their own consumption experiences and to complete the questionnaire survey accordingly. To improve response validity, only individuals who reported prior experience purchasing or using sports brand products were invited to participate (Deheshti et al., 2016). This procedure was adopted to ensure that respondents could provide valid evaluations of brand image, product quality, self-image congruence, and consumer satisfaction based on actual experience rather than hypothetical judgment. Consumers with different characteristics exhibit distinct self-image traits. Beyond common demographic variables, such as age, gender, educational level, and occupation, this study incorporates factors, such as income level, monthly sports expenditure, and weekly exercise frequency, all of which are core dimensions for assessing sports consumers’ self-image. Within this framework, we explored the relationship between the image of sports brands and CS. The questionnaire was distributed both online and offline. All participants signed informed consent forms. This study was approved by the Ethics Committee of Soochow University (No. SUDA20250527H11). Our study is also in compliance with the Helsinki Declaration.

Following data screening, 534 valid questionnaires were retained for the final analysis. The sample comprised 248 male respondents and 286 female respondents. Regarding age, participants aged 40–49 years constituted the largest group, accounting for 26.6% of the sample, followed by those aged 20–29 years at 23.2% and those aged 50 years and above at 21.2%. In terms of occupation, company employees represented the largest subgroup at 31.3%, followed by students at 21.3%. Concerning monthly personal income, the largest proportion of respondents reported an income of 5,000–8,000 yuan, accounting for 29.2%, followed by 3,000–5,000 yuan at 26.8%. In addition, 47.4% of the respondents reported spending less than 200 yuan per month on sports-related products or activities. Regarding exercise frequency, the largest proportion of participants reported engaging in physical exercise three times per week, accounting for 27.5%. Detailed sample characteristics are presented in Table 1.

**TABLE 1:** Description of the distribution of sample characteristics.

Item	Characteristic	*N*	Percentage/%
Gender	Male	248	46.4
	Female	286	53.6
Age	Under 19	57	10.7
	20–29 years old	124	23.2
	30–39 years old	98	18.4
	40–49 years old	142	26.6
	50 and older	113	21.2
Occupation	Company employee	167	31.3
	Professionals (e.g., doctors, teachers, etc.)	67	12.5
	Public servants	76	14.2
	Students	114	21.3
	Other	110	20.6
Monthly personal income	Below 3,000 yuan	131	24.5
	3,000–5,000 yuan	143	26.8
	5,000–8,000 yuan	156	29.2
	Over 8,000 yuan	104	19.5
Sports spending	Under 200 yuan	253	47.4
	200–500 yuan	158	29.6
	500–1,000 yuan	79	14.8
	Over 1,000 yuan	44	8.2
Frequency of participating in physical exercise	0 times	23	4.3
	1 time	125	23.4
	2 times	121	22.7
	3 times	147	27.5
	4 times and above	118	22.1

### 3.2 Measurement tools

#### 3.2.1 Consumer satisfaction

As the core dependent variable in the present study, CS was measured using the standardized scale (comprising five items) developed by Dwivedi (2015), Rambocas et al. (2018). These items comprehensively assess consumers’ overall perceptions of sports brands, with items such as “The sports brand meets my expectations,” “Choosing this sports brand was a wise decision,” and “I am satisfied with the sports brand.” The scale items cover the cognitive and emotional dimensions during brand selection. The measurement employed a five-point Likert scale, in which respondents indicated their level of agreement with each statement by selecting the most appropriate option (“Strongly Agree,” “Agree,” “Undecided,” “Disagree,” and “Strongly Disagree”) based on their genuine feelings. This scale design ensures data reliability and validity, facilitating subsequent analysis of CS drivers. In this study, the scale achieved a Cronbach’s alpha coefficient of 0.871, indicating strong internal consistency.

#### 3.2.2 Self-image congruence

Self-image congruence served as a key mediating variable in this study, and its measurement was based on Li et al.’s (2022) SIC Self-Consistency Scale, comprising six items. These items, which focused on examining the association between brand personality and consumers’ personal image, included statements such as “Sports brands align with how I see myself,” “My personality is highly congruent with the personality traits of sports brands,” and “My personality is very similar to that of sports brands.” The scale was measured using a five-point Likert scale, where respondents selected from five levels (“Strongly Agree,” “Agree,” “Undecided,” “Disagree,” and “Strongly Disagree”) to quantify their subjective evaluation of the brand–self-image alignment. This measurement approach helps reveal psychological mechanisms in brand selection. The Self-Image Congruence Scale demonstrated good internal consistency with a Cronbach’s alpha coefficient of 0.826.

#### 3.2.3 Brand image

Brand image, one of the independent variables was measured using the comprehensive seven-item scale recommended by Araújo et al. (2023). These items focus on the emotional, personality, and social identity dimensions of brands (e.g., “The sports brand evokes sympathy,” “The sports brand conveys a personality that makes it stand out from competitors,” and “Purchasing products from a sports brand reflects who I am”) to capture the overall image of the brand in consumers’ minds. All measurements employed a five-point Likert scale on which respondents indicated their agreement using the options “Strongly Agree,” “Agree,” “Undecided,” “Disagree,” and “Strongly Disagree.” In this study, the Cronbach’s alpha coefficient for the BI scale was 0.894, indicating good internal consistency.

#### 3.2.4 Product quality

The PQ measurement was based on the authoritative scale introduced by Yuen and Chan (2010), comprising nine items that emphasize product utility and diversity (e.g., “The sports brand offers multiple color options,” “The sports brand provides diverse material choices,” “The sports brand is practical,” and “The sports brand is fashionable”). All measurements employed a five-point Likert scale on which the respondents made their judgments using the options “Strongly Agree,” “Agree,” “Undecided,” “Disagree,” and “Strongly Disagree.” This ensured the accuracy and consistency of independent variable data, providing a solid foundation for subsequent research models. The Cronbach’s alpha coefficient for the PQ scale was 0.918, indicating high internal consistency. The construct reliabilities and average variance extracted (AVE) values are provided in Supplementary Table 1.

### 3.3 Statistical analysis

Statistical analysis was conducted using SPSS 27.0 and AMOS 24.0 software. The analysis focused on the following aspects: (1) continuous variables were represented by mean and standard deviation (M ± SD), while categorical variables were expressed by frequency; (2) Pearson correlation coefficients were used to assess the interrelationships between BI, PQ, SIC, and CS; (3) common method variance (CMV) was examined for the scales used in this study; (4) the models were tested using AMOS 24.0; and (5) AMOS 24.0 was employed to validate SIC’s mediating role between BI, PQ, and CS. Furthermore, the mediation effects were estimated using 5,000 Bootstrap resamples, with 95% CI reported. A mediation effect was considered significant if the 95% CI did not include zero.

## 4 Results

### 4.1 Descriptive statistics and correlation tests

Pearson correlation analysis was conducted to examine the relationships among gender, age, income, BI, PQ, SIC, and CS. The results are presented in Table 2, indicating that BI, PQ, SIC, and CS were all significantly correlated with one another at the 0.01 level. Based on the correlation coefficients, among BI, PQ, SIC, and CS, all pairwise correlations were positive and significant at the 0.01 level.

**TABLE 2:** Results of pearson correlation analysis between dimensions.

Variable	*M*	SD	Gender	Age	Income	BI	PQ	SIC	CS
Gender	1.54	0.499	1	–	–	–	–	–	–
Age	3.24	1.31	0.104*	1	–	–	–	–	–
Income	2.44	1.062	0.061	0.178**	1	–	–	–	–
BI	24.51	4.153	−0.039	−0.019	−0.025	1	–	–	–
PQ	31.29	5.461	−0.044	−0.006	−0.029	0.773**	1	–	–
SIC	19.63	3.395	−0.095*	−0.006	−0.013	0.683**	0.666**	1	–
CS	17.61	3.13	−0.057	−0.029	−0.112**	0.732**	0.692**	0.671**	1

Mean, standard deviation, and correlation coefficient of each variable. BI, brand image; PQ, product quality, SIC: self-image congruence, CS: consumer satisfaction. *Significant at the 0.05 level (two-tailed). **Significant at the 0.01 level (two-tailed).

### 4.2 Common method bias

Given the use of participant self-report data in this study, common method bias was assessed via Harman’s one-factor test. The analysis yielded a first factor explaining 19.84% of total variance, which is below the 40% critical threshold, indicating no severe common method bias in this study.

### 4.3 Multicollinearity diagnosis

Table 3 presents the results of the multicollinearity diagnosis among the independent variables in this study. Tolerance and the variance inflation factor (VIF) were used as evaluation criteria. Generally, if the tolerance is below 0.10 or the VIF exceeds 10, it indicates a serious multicollinearity problem.

**TABLE 3:** Multicollinearity diagnosis.

Variable	Tolerance	VIF
BI	0.352	2.844
PQ	0.367	2.723
SIC	0.486	2.058

Table 3 suggests that multicollinearity is not a problem. The tolerance values range from 0.352 to 0.486, all above 0.10, and the VIF values range from 2.058 to 2.844, all below 5.00, indicating that there is no serious multicollinearity issue among the variables, which meets the requirements for further regression analysis. Notably, the correlation between BI and PQ was relatively high (*r* = 0.773), suggesting that the distinction between the two constructs should be interpreted with caution. However, the VIF values did not exceed 5, indicating that multicollinearity was not a serious concern in the present study.

### 4.4 Structural equation model fitting indices

Structural equation modeling was conducted using AMOS 24.0 to examine the mediating role of consumer self-image between sports BI and CS. A mediation model was established with BI and PQ as independent variables, CS as the dependent variable, and SIC as the mediating variable.

Based on the model fit test results presented in Table 4, the CMIN/DF (chi-square to degrees of freedom) value is 2.793, indicating good model fit (Kline, 2015). The RMSEA is 0.058, below the 0.08 threshold and within an acceptable range (Browne and Cudeck, 1993). Furthermore, the results for the IFI, TLI, and CFI all exceed 0.9, at 0.930, 0.922, and 0.930, respectively (Hu and Bentler, 1999). Based on these findings, the results indicate that the model demonstrates good fit.

**TABLE 4:** Fit indices of the model.

Index	Reference	Observed values
CMIN/DF	1–3 indicates excellent fit, 3–5 indicates good fit	2.793
RMSEA	<0.05 is excellent, <0.08 is good	0.058
IFI	>0.9 is excellent, >0.8 is good	0.930
TLI	>0.9 is excellent, >0.8 is good	0.922
CFI	>0.9 is excellent, >0.8 is good	0.930

### 4.5 Test for the mediating effects of self-image congruence

**FIGURE 2:**
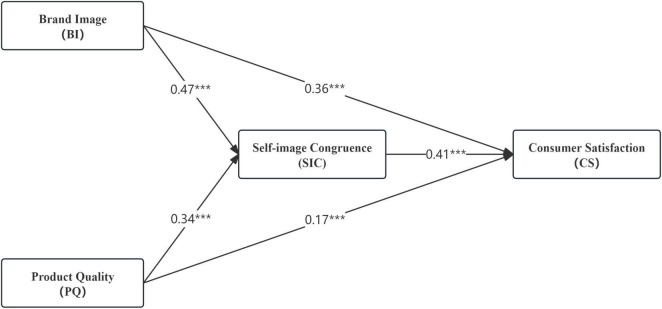
The mediating model of self-image congruence among brand image, product quality and consumer satisfaction. ***p < 0.001.

Building upon the above analysis, this study employed 5,000 bootstrap samples with a 95% confidence level, using the bias-corrected confidence interval method to test whether SIC mediates the relationship between sports BI, PQ, and CS. If the confidence interval for the mediating path coefficient does not include 0, the mediating effect is significant; otherwise, it is insignificant. The results are shown in Table 5. The hypothesized mediation model is presented in Figure 2.

**TABLE 5:** Mediated effects test results.

Hypothetical path	Effect	β	Bias-corrected 95% CI			Relative effect ratio
		Effect quantity	Lower	Upper	*P*-value	
BI→SIC→CS	Indirect effect	0.141	0.082	0.226	0.001	44.90%
	Direct effect	0.173	0.071	0.291	0.001	55.10%
	Total effect	0.314	0.214	0.425	0.001	–
PQ→SIC→CS	Indirect effect	0.192	0.108	0.300	0.001	34.66%
	Direct effect	0.363	0.205	0.510	0.001	66.34%
	Total effect	0.555	0.424	0.689	0.001	–

The test results indicate that the indirect effect of SIC on the relationship between BI and CS is significant and positive (β = 0.141, *p* < 0.001, 95% CI [0.082, 0.226]). The interval does not include zero, accounting for 44.90% of the total effect. Similarly, the direct effect of BI on CS is significant and positive (β = 0.173, *p* < 0.001, 95% CI [0.071, 0.291]). The interval also excludes zero, with the direct effect accounting for 55.10% of the total effect. This finding indicates that SIC partially mediates the relationship between BI and CS. The indirect effect of SIC between PQ and CS is significant and positive (β = 0.192, *p* < 0.001, 95% CI [0.108, 0.300]), accounting for 34.66% of the total effect. The direct effect of PQ on CS is also significant and positive (β = 0.363, *p* < 0.001, 95% CI [0.205, 0.51]), accounting for 66.34% of the effect. This finding indicates that SIC also partially mediates the relationship between PQ and CS.

In summary, this study’s results demonstrate that consumer SIC mediates the relationship between sports BI and CS and between PQ and CS.

## 5 Discussion

This study examined the relationships among brand image, product quality, self-image congruence, and consumer satisfaction in the context of sports brands. The results showed that both brand image and product quality were positively associated with consumer satisfaction. In addition, self-image congruence partially mediated the relationships between brand image and consumer satisfaction and between product quality and consumer satisfaction. These findings suggest that consumer satisfaction in sports brand contexts is shaped not only by consumers’ evaluations of symbolic and functional brand cues, but also by the extent to which these cues are perceived as congruent with consumers’ self-image.

The findings indicate that CS serves as a crucial metric for consumers’ subjective evaluation of sports BI and PQ (Bloemer and Kasper, 1995). In particular, BI exhibits a significant positive correlation with CS, thus validating H1. This finding aligns with prior research (Pradita and Sitio, 2020; Arif and Syahputri, 2021). Related research findings indicate that consumers comprehensively evaluate BI factors, such as brand reputation, corporate service and innovation capabilities, and fashionable appearance. Previous studies have shown that a favorable brand image is an important antecedent of customer satisfaction (Rusmahafi and Wulandari, 2020). Consistent with this view, the present study further supports this relationship in the context of sports brands.

In accordance with consumer expectation theory, consumers form certain expectations before and after purchasing a product, and CS fluctuates accordingly when actual experiences fall short of or exceed these expectations. Studies have also shown that positive athletic BI can surpass consumers’ original expectations for the brand, thereby enhancing satisfaction (Oliver, 1980; Schiebler et al., 2025). Conversely, a negative athletic BI exerts adverse effects on consumers (Halima et al., 2021; Açikgöz et al., 2024). Beyond this, a robust BI fosters emotional connections among consumers. In their study of globally renowned brands, Kim and Chao (2019) found that consumers’ rational perceptions and emotional attachments play a crucial role in shaping BI in consumers’ minds. In vast and uncertain consumer markets, a robust sports BI fosters emotional connections, thereby enhancing CS (Chaudhuri and Holbrook, 2001).

In the current study, PQ exhibits a significant positive correlation with CS, supporting the validity of H2, which aligns with previous research (Šugrová et al., 2017; Kencana, 2018). Specifically, consumers are likely to assess whether the functional positioning of athletic brand products aligns with their preferences, whether PQ meets standards, whether garments are comfortable to wear, and whether they offer value for money—all based on perceptions of PQ before and after purchase. When these factors meet consumers’ psychological expectations, satisfaction is directly enhanced (Dodds et al., 1991). Chi and Kilduff (2011) demonstrated in their study on casual sportswear that higher-quality, more practical, and more comfortable products are more likely to meet consumers’ psychological expectations. This, in turn, enhances consumer satisfaction and directly influences consumers’ willingness to purchase sports brand products. Wilfling et al. (2022) similarly demonstrated in their study on exercise apparel that varying functional characteristics and comfort levels significantly influence consumers’ wearing experiences, thereby underscoring the critical role of reliable PQ in CS. Positive PQ not only elevates consumers’ perceived value of a product but also makes consumers dependent on the product, thereby increasing overall satisfaction (Fan and Dong, 2021; Religia et al., 2024). When PQ falls short of consumer expectations, or when a product’s functional attributes fail to meet emotional needs, this undermines CS (Shabbir, 2020).

The present study confirms that consumer SIC partially mediates the relationship between BI and CS, accounting for 44.90% of the total effect, thus validating H3. Goh et al. (2016) demonstrated in their smartphone purchase study that aligning consumers’ self-concept with smartphone BI enhances post-purchase satisfaction through increased SIC. From a sports brand perspective, when consumers perceive a strong sports BI, they actively align their self-image with BI. High consistency between BI and consumer self-image enhances brand trust, indicating that BI design aligned with target consumers’ self-perceptions elevates brand evaluations. This is supported by the literature, which shows that when sports BI strongly aligns with a consumer’s self-image, it fosters a sense of identification, delivering self-satisfaction and self-affirmation (Sirgy, 1985; Lu and Xu, 2015).

This study confirms that consumer SIC partially mediates the relationship between PQ and CS, accounting for 34.66% of the total effect, thus validating Hypothesis H4. Klabi (2020), in an investigation of international brand localization, argued that consumers’ direct experience with brand products constitutes perceived quality. The study emphasizes that perceived quality is a rationally guided behavior, relying more heavily on cognitive processing, such as reflection, imagination, and curiosity—derived from actual product experiences. The higher the frequency of this cognitive processing, the more pronounced the enhancing effect of SIC on perceived quality.

In summary, aligned with previous literature, the findings of this study reaffirm the mediating role of consumer SIC between BI and CS, as well as between PQ and CS. When distinct BIs and PQ attributes align strongly with consumers’ self-perceptions, satisfaction gains become more pronounced. In contrast, negative brand perceptions or subpar quality erode consumer satisfaction in sports brands.

### 5.1 Theoretical implications

This study provides important theoretical implications for research on sports brand consumption. First, it incorporates self-image congruence (SIC) into the brand image–product quality–consumer satisfaction framework, thereby extending the explanatory pathway of consumer satisfaction in the sports brand context. More specifically, the findings suggest that consumer satisfaction is influenced not only by brand image and product quality, but also by the extent to which consumers perceive these brand-related cues as consistent with their self-image. In this way, the study highlights SIC as an important psychological mechanism linking external brand-related cues with consumers’ satisfaction evaluations. Therefore, the present study enriches the literature on consumer behavior and brand management by providing a more integrated understanding of how symbolic and functional dimensions jointly shape consumer satisfaction.

### 5.2 Practical implications

This study also offers meaningful practical implications for sports brand managers. By catering to the self-image of target consumer groups and enhancing the consistency between brand image and consumer self-perception, sports brands may encourage consumers to select products that better match their own image traits, thereby maximizing the positive impact on consumer satisfaction. In addition, product quality should not only satisfy consumers’ fundamental practical demands, such as comfort, durability, and reliability, but also meet emotional needs by aligning perceived quality with identity value. When products meet both utilitarian and emotional requirements, consumers are more likely to develop stronger identification with the product and higher satisfaction. SIC also helps consumers translate the objective quality of sports products into attributes that are personally relevant and meaningful during product evaluation and use. Therefore, sports brands should tailor brand image to specific consumer segments, strengthen product reliability and functional attributes, and clearly communicate these qualities to consumers. Such efforts may enhance self-image congruence and further improve consumer satisfaction.

## 6 Limitations

This study has several limitations. First, the sample was drawn mainly from a specific region, which may limit the generalizability of the findings. Second, the study did not further distinguish different types of consumer self-image or conduct more refined segmentation of consumer groups. Third, although gender, age, and income were included in the analysis, these demographic variables were not discussed in depth. Fourth, the study examined sports brands at a general level and did not focus on specific brands.

Future research may address these limitations by using more diverse samples, examining different self-image profiles and demographic groups in greater depth, and testing the proposed relationships in the context of specific sports brands.

## 7 Conclusion

The findings reveal significant positive correlations between athletic BI and PQ on the one hand, and CS on the other hand. Furthermore, consumer SIC mediates both the relationship between athletic BI and CS, as well as the relationship between athletic PQ and CS. Additionally, different athletic BIs and product qualities elicit varying perceptions and mental representations among consumers, subsequently influencing their purchasing behaviors and post-consumption satisfaction. During the purchase decision stage, consumers typically screen and evaluate products from different brands based on their self-image. They tend to choose brands that align with their self-image, ensuring consistency between their self-image positioning, the brand’s image positioning, and the product’s quality/functional positioning. This consistency not only enhances CS but also strengthens their identification with and sense of belonging to the brand. Today, as consumer demand for personalization continues to grow, the role of consumer self-image in BI formation and satisfaction enhancement becomes increasingly significant. Therefore, sports brands should pay greater attention to this trend. By adopting precise market positioning and differentiated product strategies, they can shape BIs that strongly align with consumer self-image, enabling them to better meet consumer needs and enhance their purchase satisfaction.

## Data Availability

The raw data supporting the conclusions of this article will be made available by the authors, without undue reservation.
