# Supplementary material for: The impact of sports brand image and product quality on consumer satisfaction: the mediating effects of self-image congruence
Source: Front Psychol. 2026 Jul 13;17:1778226. doi: 10.3389/fpsyg.2026.1778226 (PMC13402415; doi:10.3389/fpsyg.2026.1778226)
Supplement: Supplementary file 1 [file Table_1.docx]

| Supplementary table1 Construct reliabilities and AVE. | | | | |
| --- | --- | --- | --- | --- |
| Constructs | Items | Estimate | AVE | CR |
| Brand Image | BI7 | 0.728 | 0.5483 | 0.8944 |
|  | BI6 | 0.802 |  |  |
|  | BI5 | 0.757 |  |  |
|  | BI4 | 0.74 |  |  |
|  | BI3 | 0.745 |  |  |
|  | BI2 | 0.743 |  |  |
|  | BI1 | 0.661 |  |  |
| Prodcut Quality | PQ9 | 0.768 | 0.5568 | 0.9186 |
|  | PQ8 | 0.742 |  |  |
|  | PQ7 | 0.789 |  |  |
|  | PQ6 | 0.75 |  |  |
|  | PQ5 | 0.729 |  |  |
|  | PQ4 | 0.786 |  |  |
|  | PQ3 | 0.758 |  |  |
|  | PQ2 | 0.72 |  |  |
|  | PQ1 | 0.666 |  |  |
| Self-Image Consistency | SIC1 | 0.763 | 0.5447 | 0.8772 |
|  | SIC2 | 0.791 |  |  |
|  | SIC3 | 0.805 |  |  |
|  | SIC4 | 0.687 |  |  |
|  | SIC5 | 0.668 |  |  |
|  | SIC6 | 0.703 |  |  |
| Customer satisfaction | CS1 | 0.659 | 0.501 | 0.8336 |
|  | CS2 | 0.709 |  |  |
|  | CS3 | 0.696 |  |  |
|  | CS4 | 0.737 |  |  |
|  | CS5 | 0.735 |  |  |

Table 1 shows acceptable reliability and convergent validity. All factor loadings range from 0.659 to 0.805, the CR values range from 0.8336 to 0.9186, and the AVE values range from 0.501 to 0.5568. Since all CR values exceed 0.70 and all AVE values are above 0.50, the constructs meet the recommended standards.
